# The role of pregnancy associated plasma protein-A in triple negative breast cancer: a promising target for achieving clinical benefits

**DOI:** 10.1186/s12929-024-01012-x

**Published:** 2024-02-23

**Authors:** Arpita Poddar, Farah Ahmady, Sushma R. Rao, Revati Sharma, George Kannourakis, Prashanth Prithviraj, Aparna Jayachandran

**Affiliations:** 1 Fiona Elsey Cancer Research Institute, Victoria, Australia; 2 Federation University, Victoria, Australia; 3 RMIT University, Victoria, Australia; 4 Adelaide Medical School, University of Adelaide, Adelaide, South Australia Australia

**Keywords:** Pregnancy associated plasma protein-A, Breast cancer, Triple negative breast cancer, Epithelial-mesenchymal transition, Cancer therapy, Cancer biomarker, Immunotherapy

## Abstract

Pregnancy associated plasma protein-A (PAPP-A) plays an integral role in breast cancer (BC), especially triple negative breast cancer (TNBC). This subtype accounts for the most aggressive BC, possesses high tumor heterogeneity, is least responsive to standard treatments and has the poorest clinical outcomes. There is a critical need to address the lack of effective targeted therapeutic options available. PAPP-A is a protein that is highly elevated during pregnancy. Frequently, higher PAPP-A expression is detected in tumors than in healthy tissues. The increase in expression coincides with increased rates of aggressive cancers. In BC, PAPP-A has been demonstrated to play a role in tumor initiation, progression, metastasis including epithelial-mesenchymal transition (EMT), as well as acting as a biomarker for predicting patient outcomes. In this review, we present the role of PAPP-A, with specific focus on TNBC. The structure and function of PAPP-A, belonging to the pappalysin subfamily, and its proteolytic activity are assessed. We highlight the link of BC and PAPP-A with respect to the IGFBP/IGF axis, EMT, the window of susceptibility and the impact of pregnancy. Importantly, the relevance of PAPP-A as a TNBC clinical marker is reviewed and its influence on immune-related pathways are explored. The relationship and mechanisms involving PAPP-A reveal the potential for more treatment options that can lead to successful immunotherapeutic targets and the ability to assist with better predicting clinical outcomes in TNBC.

## Introduction

**Fig. 1:**
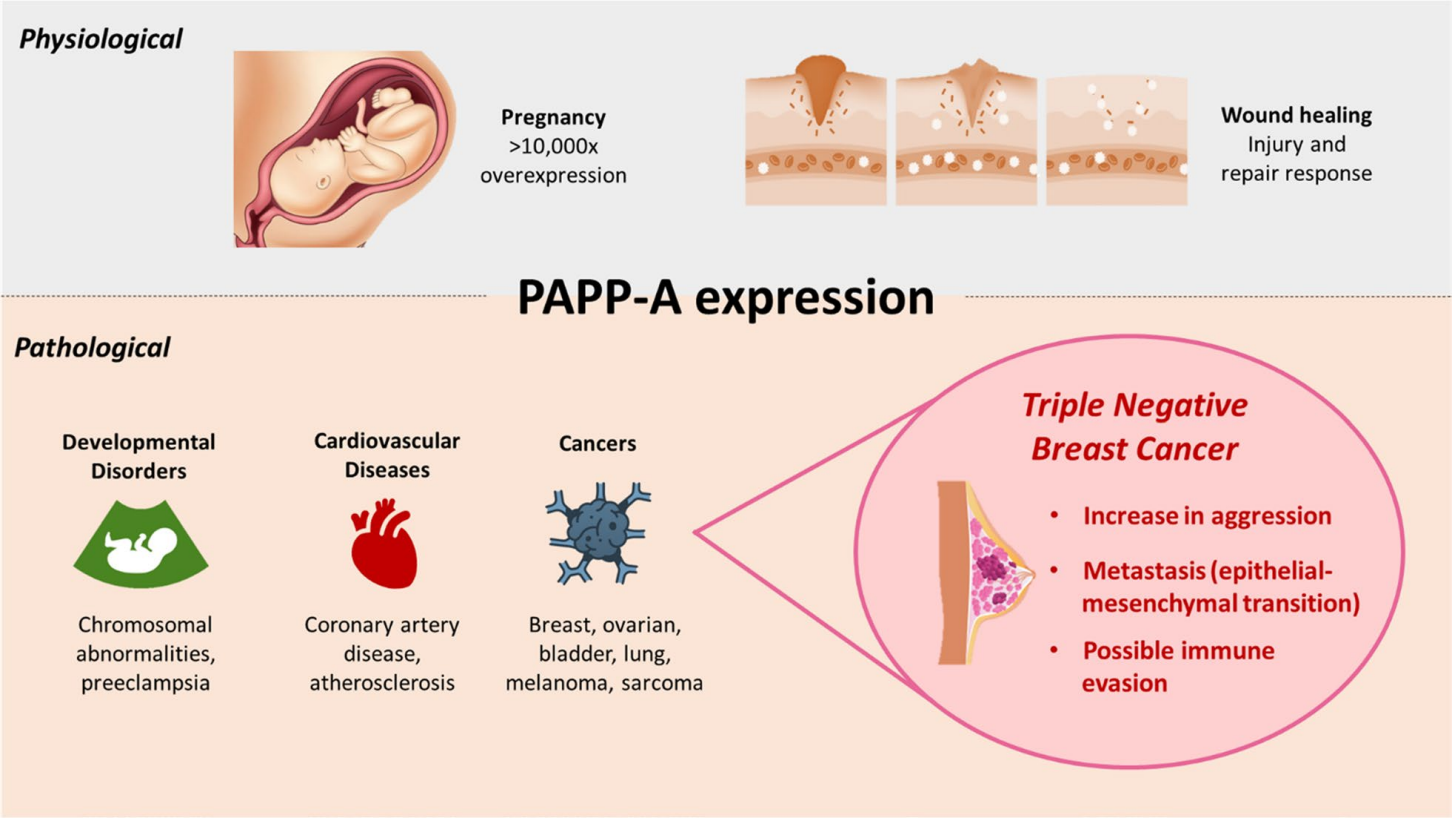
Pathological expression of pregnancy associated plasma protein-A (PAPP-A) correlates with triple negative breast cancer (TNBC)

Pregnancy associated plasma protein-A (PAPP-A) is a protein originally detected in 1974 in high quantities in the plasma of third-trimester pregnant women. Along with three other placental antigens identified, PAPP-A was found to be susceptible to proteinases and the molecular weight was estimated to be 750,000 MW at the time of initial discovery [1]. The function of PAPP-A remained unknown till its role in proteolytic activity of insulin-like growth factor (IGF) -dependent insulin-like growth factor-binding proteins (IGFBP) was revealed for the first time in cell culture media conditioned by human fibroblasts; where PAPP-A was detected and sequenced through mass spectroscopy and identified as the protein causing IGFBP-4 proteolysis [2]. It has since been found that during pregnancy, the placental syncytiotrophoblasts synthesize and secrete PAPP-A into the plasma and maintain its high levels (> 10,000 fold in humans [3]) throughout gestation [4, 5]. Subsequently, in addition to conditions not related to pregnancy, PAPP-A has been discovered to be expressed during injury and repair responses for wound healing, cardiovascular and developmental diseases, and several malignancies, albeit in smaller concentrations than the placenta (Fig. 1) [5, 6]. PAPP-A is a metalloproteinase with IGFBP-4 proteolytic activity now demonstrated in breast, ovarian, lung, smooth muscle, bone, kidney, thymus, adipose, cardiovascular and immune cells; and its deletion has been implicated in promoting longevity and reducing tumor burden [7–11]. PAPP-A cleaves three of the six IGFBPs known, such as IGFBP-4, leading to increased bioavailability of IGFs, which in turn mediates cell proliferation, migration and survival [12]. High quantities of IGFs and the enhanced type 1 insulin-like growth factor receptor (IGF-IR) signaling pathways are heavily implicated in tumor initiation and progression [6, 13, 14].

Heightened expression of PAPP-A is seen across various cancer types, including breast, ovarian, lung, prostate, pancreatic, liver, and thyroid cancers, as well as uterine leiomyomas, Ewing sarcoma, mesothelioma, and melanoma [15]. Numerous cell culture studies and mouse xenograft models have substantiated the oncogenic role of PAPP-A [16–18]. In addition to increased PAPP-A protein levels seen in ascites of ovarian cancer and pleural effusions of mesothelioma, PAPP-A has also been detected in the tumor microenvironment as tumor promoting stroma-secreted factor in hepatic stellate myofibroblasts and in cancer associated fibroblasts [19–22]. The role of PAPP-A in tumor behavior is apparent; however, despite the known link, very little information is available about its role specifically in human breast cancer (BC). Indeed, the link between PAPP-A and BC was reported even prior to the discovery of PAPP-A function and its involvement in the IGF system; wherein independent of estrogen receptor (ER) status in stage I BC, PAPP-A was found to be a clinically significant predictor of early recurrence [23]. It has since been reported that PAPP-A is overexpressed in more than 70% of BC [24]. In this review, we take a comprehensive look exclusively at the role of PAPP-A in BC, focusing on the aggressive subtype of triple negative breast cancer (TNBC). We present a complete picture of PAPP-A activity that has emerged so far in TNBC, with specific emphasis on the functional regulation of PAPP-A that causes TNBC progression and metastasis, its relation to the immune system, as well as its clinical relevance. Systematically, this review covers a guide to PAPP-A structure, function, and regulation – critical for understanding the features important for TNBC therapy and biomarker selection ("PAPP-A: structure, function, and regulation" section); a comprehensive summary of the biological link with BC including TNBC till date – the IGFBP and the IGF axis, epithelial-mesenchymal transition, the window of susceptibility, and pregnancy ("PAPP-A: specificity in BC subtypes" section); influence on immune-related pathways – potential for immunotherapy in TNBC ("PAPP-A in TNBC: proteolysis of IGFBPs" section); and an assessment of clinicopathological relevance of PAPP-A in TNBC ("Clinical relevance of PAPP-A in TNBC" section). Altogether, a comprehensive exploration of potential pathways for future TNBC research is presented by placing the role of PAPP-A within the context of physiological processes that impact BC inititation, progression and metastatic spread.

One in seven women are at the risk of BC in their lifetime and it is one of the leading causes of cancer-related mortality and morbidity in women [25]. Globally, it is the most commonly diagnosed cancer and is the fifth leading cause of death [26]. BC is sub-divided into four subtype categories according to the hormone receptor status: Luminal A (ER + , progesterone receptor (PR) + , and human epidermal receptor 2 (HER2)-); Luminal B (ER + and/or PR + , HER2 +); HER2 overexpressing (ER-, PR- and HER2 +); and triple negative (ER-, PR- and HER2-) [27]. Among these subtypes, TNBC is the most aggressive, is frequently diagnosed at earlier ages and has median survival of < 14 months once metastasized [28, 29]. For non-TNBC subtypes, the 5-year survival rate is 99% and women diagnosed with this disease have several successful therapies such as hormonal and anti-HER2 targeted treatments [30]. However, TNBCs, comprising 15–20% of all BC cases, lack targeted therapy, have much poorer clinical outcomes, show increased local and distant recurrences, and have significantly lower survival rates [31, 32]. TNBC phenotype is highly prevalent in women diagnosed with BC during pregnancy and is associated with a poor prognosis [27]. This aggressiveness of BC is reported to link with the highly elevated levels of PAPP-A throughout pregnancy. Increased PAPP-A expression in BC cells have been shown to correlate with tumor progression through epithelial-mesenchymal transition (EMT), and clinically tends towards worse overall survival [16]. However, while emerging studies make it clear that the IGFBP cleaving enzyme PAPP-A is heavily implicated in breast tumorigenesis, a few differential results report PAPP-A as having a more tumor suppressive role. For instance, PAPP-A silencing has led to an increase in BC aggressiveness; and a reduction in maternal serum concentrations of PAPP-A was found to correlate with elevated BC risk [33, 34]. Such studies, explored in detail further in the review, provide insights that PAPP-A needs to be more thoroughly investigated. Tumor staging and cancer subtypes account for the conflicting observations in part. Nonetheless, detailed assessments are necessary to resolve the mechanisms and impacts of PAPP-A action in mammary tumorigenesis in order to fully realize its promising potential as a therapeutic and/or diagnostic target in TNBC.

Functionally, PAPP-A proteolyzes three IGFBPs, causing highly specific, regulated and efficient cleavage of IGFBPs-4, -5 and -2 at single sites. IGFBPs-4 and -2 cleavages require prior IGF-I or IGF-II binding before PAPP-A activity, and IGFBP-5 degradation is IGF independent [7]. PAPP-A interacts and binds with the cell surface glycosaminoglycans to become active for proteolysis of the IGFBPs [35]. Following proteolysis, the free IGFs bind to their respective receptors and initiate downstream signaling leading to enhanced proliferation, metastasis and survival [36, 37]. Some regulators of PAPP-A have been reported, such as inhibition by proform of the eosinophil major basic protein (proMBP) and stanniocalcins STC1 and STC2 [38–40]. Furthermore, PAPP-A overexpression has recently been shown to significantly promote migration, invasion and EMT, especially in BC cells [16, 17]. This review highlights the current research that lays the groundwork for evaluating PAPP-A as a biomarker, treatment modality or diagnostic molecule in BC – elucidating its involvement specifically in TNBC, highlighting the structure and function of PAPP-A, its regulators, and links to tumor progression. Importantly, we provide insights on its correlations with the immune system, its clinical applications for BC prognosis and potential in therapeutic targeting.

## PAPP-A: structure, function, and regulation

### Structure

The gene for PAPP-A is located on chromosome 9q33.1 in humans, comprising 22 exons and 21 introns [41]. Highly conserved among species, PAPP-A shares more than 90% homology between the human and murine proteins [42]. Interestingly though, unlike humans, placenta of rats and mice do not produce PAPP-A [43]. The preprocessed protein is a 1627-residue polypeptide that undergoes maturation to yield a 1547 kDA protein [44]. In non-pregnancy related states and tissues, PAPP-A is a 400 kDA homodimer [45]. In pregnancy plasma, PAPP-A was initially thought to be a tetramer comprising approximately 200 kDA subunits [45]. However, it has since been shown to be a heterotetrametric complex made of two separate equimolar chains linked with disulfide bridges [46]. One chain is comprised of two PAPP-A subunits and the other chain is comprised of two disulfide bridged subunits of proMBP [45]. The PAPP- A/proMBP subunits are glycoproteins consisting of carbohydrates, glycosaminoglycans, zinc binding motifs, metal chelation columns and heparin binding sites [47]. Free proMBP is the precursor of eosinophil major basic protein (MBP) and is cleaved to release cytotoxic MBP during maturation of the eosinophil precursor cells [48]. However, there are no reports observed for proMBP of the PAPP-A/proMBP complex to go through a similar process. In the complexed form, proMBP functions as an inhibitor of the proteolytic activity of PAPP-A, reducing PAPP-A proteinase activity by more than 100-fold. Trace amounts of less than 1% uncomplexed PAPP-A are also present in pregnancy plasma and serum [46].

The PAPP-A subunit contains a 250-residue laminin G-like module at the N-terminus, the function of which is so far unknown [49]. It is followed by the elongated zinc binding protein motif consensus sequence HEXXHXXGXXH (His-482-His-492) of 350-residues [50]. Presence of this consensus motif places PAPP-A in the metzincin superfamily of metalloproteinases [51], where it is the founding member of the subgroup called pappalysins. A homologue of PAPP-A has been identified, termed PAPP-A2, that is also a pappalysin and is estimated to have similar functions in growth regulation as PAPP-A [52]. Three linear notch repeat (LNR) sequences are contained in the subunit, two of which span the proteolytic domain of PAPP-A close to the N-terminus and the third is towards the C terminus [53]. The proteolytic domain consists of roughly 350 residues and the Glu-483 is critical for catalytic activity [50]. Five complement control protein (CCP) modules are present close to the C terminus that contain glycosaminoglycan binding sequences to facilitate PAPP-A cell surface attachment [35, 54].

During human pregnancy, serum PAPP-A/proMBP levels continually increase up to parturition [55]. The syncytiotrophoblasts are the main source of PAPP-A and extravillous X cells or cytotrophoblasts are the source of proMBP [4]. At term, up to 50 mg/L of serum PAPP-A circulates as PAPP-A/proMBP [56]. This covalent complex is established in the extracellular environment and accounts for nearly 99% of circulating PAPP-A. However, in the first trimester, up to approximately 30% can be in the form of uncomplexed PAPP-A [57]. Regardless of cancer, since the early 90 s, aberrant levels of PAPP-A have been known to indicate unfavorable pregnancy outcomes such as Down’s syndrome, low birth weights, preeclampsia, growth retardations and other chromosomal abnormalities [58–60]. In males, immunohistochemistry (IHC) studies have revealed PAPP-A presence in Leydig cells, epididymis, testes and seminal vesicles as well as semen. Abnormally high levels of circulating PAPP-A levels correlated with prostate and testicular cancers, and levels decreased following orchidectomy or prostatectomy [61].

### Function

The primary function of PAPP-A is proteolysis (Table 1), despite the presence of the metalloproteinase domain [37]. The first demonstration of PAPP-A proteolysis was IGFBP-4 cleavage [2]. Antibodies against PAPP-A blocked IGFBP-4 degradation in media conditioned with human fibroblasts. Pregnancy serum PAPP-A cleaved IGFBP-4 and secreted PAPP-A proteins were isolated from fibroblasts and osteoblasts [2]. Since then, PAPP-A mediated IGFBP-4 proteolysis has been seen in ovarian, lung, smooth muscle and endometrial stromal cells [62]. A distinctive feature of IGFBP-4 cleavage is the requirement of IGF-I or IGF-II to be present [63]. IGF-II is reported to be more efficient than IGF-I, and their binding to IGFBP-4 enhances sensitivity to PAPP-A. PAPP-A subsequently cleaves the IGFBP-4 protein at the sites Met-135 and Lys-136, thus releasing the bound IGFs and increasing their bioavailability for activating IGF receptor specific downstream signaling pathways [63]. Such PAPP-A activity can proceed in an autocrine or paracrine manner [35]. A small proportion of IGF-II independent IGFBP-4 cleavage by PAPP-A is also recorded, possibly at the same site, although proceeding at much lower rates of kinetic efficiency than in the presence of IGFs [63]. A comparison study utilizing a recombinant PAPP-A protein expressed from human embryonic kidney 293T cells identified a single nucleotide polymorphism (SNP) in the PAPP-A allele (rs7020782; serine < tyrosine) that effects the proteolytic cleavage of IGFBP-4 [64]. The SNP with the serine variant was found to be significantly less efficient for cleaving IGFBP-4 as compared to the tyrosine variant. However, impact of the SNP on other IGFBP substrates such as IGFBP-5 and IGFBP-2 was not significant [64].

**Table 1:** Known PAPP-A mediated IGFBP proteolysis

IGFBP	IGF requirement	Role of PAPP-A	Kinetic efficiency	References
IGFBP-4	IGF-II (more efficient) or IGF-I	Cleavage at Met-135 and Lys-136	High	[63]
IGFBP-4	Not required	*Same site	Very low	[63]
IGFBP-5	Not required; inhibited by IGF presence	Cleavage at Ser-143 and Lys-144	High	[65, 66]
IGFBP-2	IGF dependent	Cleavage at Gln-165 and Met-166	Less than IGFBP-4	[67]

*Possibly; recorded less than IGF dependent

IGFBP-5 is another substrate for PAPP-A. Like IGFBP-4, proteolysis occurs roughly in the middle of the protein sequence (between Ser-143 and Lys-144 of IGFBP-5) to yield similar sized degraded fragments. But unlike IGFBP-4, PAPP-A mediated IGFBP-5 cleavage requires the presence of a 25-amino acid anchor peptide, and it is not only IGF independent but also found to be inhibited in the presence of IGFs by nearly three-fold [65, 66, 68]. PAPP-A mediates cleavage of IGFBP-2 as well [67]. IGFBP-2 is less susceptible than IGFBP-4 to PAPP-A, but its hydrolysis is also IGF-dependent, occurring between Gln-165 and Met-166. Similar to activity with IGFBP-4, IGFs are not considered as directly interacting with PAPP-A, rather, IGF binding to the IGFBPs-2 or -4 render conformational changes that enhance the degradation process [67]. Once IGFs are liberated from the IGF/IGFBP complexes, the IGFs bind to IGF receptors and cause initiation of multiple signaling pathways, leading to enhancement in cell proliferation and migration capacities, as well as reduced cell death and apoptosis [69]. This fosters a tumorigenic environment and promotes carcinogenesis of tissues, with PAPP-A therefore playing a critical role in establishment of malignancy.

### Regulation

Several regulators of PAPP-A activity are emerging (Table 2). The first known inhibitor of PAPP-A is proMBP [46]. proMBP covalently binds PAPP-A and almost completely abrogates any proteolytic activity [70]. So far, all reported proteolytic function of PAPP-A is found in tissues where it is present free of proMBP [70]. As the levels of serum PAPP-A as PAPP-A/proMBP complex are increased by more than 10,000-fold during pregnancy, it stands to reason the strong inhibitory action of proMBP on PAPP-A protease activity has a major role in preventing PAPP-A mediated tumorigenesis during normal physiology. Stanniocalcins (STC) 1 and 2 are mammalian glycoprotein hormones that also potently inhibit PAPP-A, and both are thought to be proteinases specific to pappalysins [39, 40, 71]. STC1 is abundantly synthesized in multiple organs including heart, lung, liver, kidney, adrenal gland, ovary and prostate. Although a consensus role of STC1 is lacking, it is proposed to be associated with tumorigenesis as well as other physiological pathways of chondrogenesis and adipogenesis [39]. STC1 has been shown to interact with PAPP-A with strong affinity, but not with covalent binding, and inhibits PAPP-A proteolysis of IGFBP-4 and is antagonistic towards PAPP-A facilitated phosphorylation of IGF-IR [39]. The potent inhibitory effect of STC2 on PAPP-A activity is reported to occur through covalent binding of the cysteine-120 residue of STC2 with PAPP-A [40]. This leads to prevention of IGFBP-4 proteolysis by PAPP-A and the subsequent increased bioavailability of IGFs in tissues. Interestingly, mice overexpressing wild-type STC2 showed growth retardation, whereas mice overexpressing mutated STC2 that could not inhibit PAPP-A did not show any growth retardation [40]. Similarly, PAPP-A knockout mice are smaller in size compared to their wildtype littermates and have longer life span [14]. Such results suggest that PAPP-A inhibition can reduce cell growth in vivo.

**Table 2:** Regulators of PAPP-A activity

Regulator	Role	Mode of PAPP-A interaction	Organism	References
proMBP	Inhibitor	Covalently binds PAPP-A to abrogate its proteolytic activity	Human serum, HEK 293T Cells	[46, 70]
STC1	Inhibitor	High affinity binding to PAPP-A rather than covalent binding	HEK 293T Cells	[39]
STC2	Inhibitor	Covalent binding of PAPP-A through Cys-120 residue of STC2	HEK 293T Cells, transgenic mice, mouse embryonic fibroblasts	[39, 40]
cAMP	Inducer	cAMP inducible region in 5’ UTR of PAPP-A cDNA	Human placental choriocarcinoma cell line JAR cells	[72]
Progesterone antagonist (RU486)	Inhibitor	Inhibition of PAPP-A production rate; PAPP-A production recovered by addition of progesterone	Human trophoblastic and decidual explants, cynomolgus monkey	[73, 74]
PMSG	Inducer	Transient increase in PAPP-A transcripts	Mouse ovary	[74]
hCG	Inducer	Sustained increase in PAPP-A expression after PMSG treatment	Mouse ovary	[74]
FSH	Inducer	Increased PAPP-A mRNA expression	Rat granulocytes	[76]
BMP-15	Inhibitor	Reduced PAPP-A expression following FSH stimulation	Rat granulocytes	[76]
p53	Inhibitor or inducer	PAPP-A suppression in TNBC; PAPP-A overexpression in human fibroblasts	TNBC cell line MDA-MB-157; BJ/ET cell line	[81–83]
Bikunin	Inhibitor	Early suppression of PAPP-A mRNA in response to bikunin treatment	Ovarian cancer cell line HRA	[79]
TNF-α, IL-1β, IL-6, IL-4, TGF-β	Inducer	Upregulation of PAPP-A expression	TNF-α and IL-1β: human dermal fibroblasts and human coronary artery endothelial and smooth muscle cells; IL-6: coronary artery smooth muscle cells; TNF-α, IL-1β**,** IL-4, TGF-β: human osteoblasts	[78]
EGF	Inducer	Upregulation of PAPP-A expression	TNBC cell lines	[16]
INF-γ	Inhibitor	Suppression of PAPP-A expression	Human fibroblasts	[80]
Resveratrol	Inhibitor	Reduction in cytokine-mediated PAPP-A expression	Coronary artery smooth muscle cells	[80]
miRNA-214	Inhibitor	Targeted suppression of PAPP-A mRNA	NSCLC cell lines U-1810 or H23	[84]
miR-497-5p	Inhibitor	Negative regulator of PAPP-A mRNA	Pregnancy-associated BC tissues and serum, normal breast tissues, BC cell lines MDA-MB-231 and MCF-7	[17]
miR-490-3p	Inhibitor	Targeted suppression of PAPP-A expression	Human coronary artery smooth muscle cells	[85]
miR-141	Inhibitor	Suppression of PAPP-A protein	Vascular smooth muscle cells	[86]

Cyclic adenosine monophosphate (cAMP) is another reported regulator of PAPP-A expression. Cloning and sequencing of the PAPP-A cDNA revealed a long 5’ untranslated region (5’ UTR) that bears a cAMP inducible region, and PAPP-A protein synthesis could be induced in vitro in the presence of cAMP [72]. PAPP-A has also been reported to be progesterone dependent, where in vitro treatment with progesterone antagonists depleted PAPP-A secretion and subsequent progesterone treatment recovered PAPP-A expression in humans and monkeys [73, 74]. Hormonal regulation of PAPP-A during the estrous cycle has been seen in a comparison study of human PAPP-A with the mouse ovarian PAPP-A cDNA [75]. Sequencing of PAPP-A from the two species demonstrated 88% match at the nucleotide level, 89% at the amino acid level and 93% similarity score for conservative amino acid substitutions. While PAPP-A transcripts in mice ovaries could not be detected to the same extent as human placenta and human fibroblasts, injection with pregnant mare serum gonadotropin (PMSG) led to transient increase in levels of PAPP-A transcripts in the mouse ovarian tissues at specific locations in granulosa and follicles. Subsequent treatment of the above mice with human chorionic gonadotropin (hCG) reintroduced sustained PAPP-A expression from ovulation to luteinization [75]. PMSG, as well as follicle stimulating hormone (FSH) also induce PAPP-A mRNA expression in granulocyte cells of rats in a spatiotemporal manner [76]. The oocyte growth factor, bone morphogenetic protein (BMP)-15, is reported to inhibit PAPP-A expression that has been enhanced by FSH [76]. Such PAPP-A expression is noted to occur at significantly differential patterns in rhesus monkeys [77].

Pro-inflammatory cytokines including tumor necrosis factor (TNF)-α, interleukin (IL)-1β and IL-6 have been seen as strong inducers of PAPP-A expression in human dermal fibroblasts and human coronary artery endothelial and smooth muscle cells; with IL-6 mediated PAPP-A upregulation seen only in the coronary artery smooth muscle cells [78]; and TNF-α, IL-1β, IL-4 and transforming growth factor-β (TGF-β) induced expression is seen in human osteoblasts as well [78]. In line with such effects of pro-inflammatory cytokines, a Kunitz-type protease inhibitor called Bikunin, responsible for suppression of pro-inflammatory cytokines, has been reported to suppress PAPP-A expression. Bikunin overexpression in human ovarian cancer cell line could decrease cancer invasion and metastasis, and acted as a suppressor of the PAPP-A gene with a nearly ninefold reduction in PAPP-A RNA. In the study, knockdown of PAPP-A led to decrease in invasiveness of the ovarian cancer cells [79]. Resveratrol, another anti-inflammatory molecule, has also been shown to reduce cytokine-mediated PAPP-A expression in coronary artery smooth muscle cells, and interferon-γ (INF-γ), another cytokine with both pro and anti-inflammatory roles, has reduced PAPP-A expression in human fibroblasts [80]. PAPP-A thus appears to be involved more in promotion of inflammation than inflammation suppression responses. However, such a feature is not conclusive, as some of the cytokines have dual impacts on inflammation. In addition to cytokines, growth factors such as the epidermal growth factor (EGF) are reported to induce PAPP-A expression. Importantly for BC, in the TNBC cell line MDA-MB-468, treatment with EGF increased PAPP-A expression. The study highlighted the role of EGF as a potent activator of EMT and the resultant PAPP-A expression correlated strongly with increase in mesenchymal markers [16]. As discussed before, in a study in human osteoblasts, TGF-β also induced PAPP-A expression [78]. TGF-β is another well know inducer of EMT, potentiating further indications of the link between PAPP-A and EMT; explored in more details in the review in "PAPP-A in TNBC: role in Epithelial-Mesenchymal Transition (EMT)" section. The reported role of the tumor suppressor p53 as a regulator of PAPP-A in BC is also explored further in “PAPP-A in TNBC: proteolysis of IGFBPs” section. The wild type protein could lead to PAPP-A suppression in TNBC cell line MDA-MB-157, but in human fibroblasts, p53 appeared to cause overexpression of PAPP-A [81–83].

Furthermore, microRNA (miRNA) mediated alteration of PAPP-A expression have also been reported. miRNA-214, a metastasis-linked mediator, is a potential suppressor of PAPP-A. Knockdown of miRNA-214 in non-small cell lung carcinoma cell lines upregulated PAPP-A expression, and overexpression of miRNA-214 decreased PAPP-A expression [84]. In human coronary artery smooth muscle cells, miR-490-3p has been demonstrated to target PAPP-A and inhibit its upregulation. The resultant downregulated PAPP-A led to a decrease in IGFBP-4 proteolysis. Conversely, inhibition of miR-490-3p upregulated PAPP-A expression and increased its proteolytic activity on IGFBP-4 [85]. In vascular smooth muscle cells, miR-141 is reported to repress PAPP-A expression by directly inhibiting its translation. Interestingly, PAPP-A protein, and not PAPP-A mRNA, appeared to be significantly reduced following overexpression of miR-141 [86]. Importantly for BC, in BC cell lines MDA-MB-231 and MCF-7, miR-497-5p is found to be a negative regulator of PAPP-A. MiR-497-5p could significantly reduce PAPP-A expression in BC cell lines and serum of pregnancy associated BC patients as compared to non-cancer tissues [17].

To summarize, it is evident that not all regulators of PAPP-A have been discovered or validated in BC. It must be noted that, to the best of our knowledge, not many similar studies have been carried out to assess for PAPP-A regulation specifically in human mammary glands. The presence of species-based and tissue-based differences have clearly emerged, and bear consideration while assessing the biological role of PAPP-A.

## PAPP-A: specificity in BC subtypes

PAPP-A is overexpressed in nearly all subtypes of malignant BC, with luminal B correlating with higher expression than luminal A subtypes [24]. A landmark study on formalin-fixed paraffin-embedded (FFPE) tumor tissues from 46 patients with invasive BC revealed positive PAPP-A protein staining in 98% of the samples, with significantly higher detection in luminal B than luminal A. Interestingly, within the luminal B subtypes, no significant difference in PAPP-A expression could be observed between HER2- and HER2 + cases, between ER- and ER + cases, as well as between tumor grades. All TNBC specimens correlated with weak to strong positivity for PAPP-A expression. Nearly complete absence of expression was seen in non-tumor tissues. Previously, in stage II BC or stage I ER-BC, positive PAPP-A immunostaining was seen as a predictor of early recurrence [23, 87]. However, the widespread nature of PAPP-A expression in all the BC specimens did not allow for any conclusive links between intensity of PAPP-A staining and BC subtype in the studies. Nonetheless, the overall trend was towards the presence of PAPP-A associating with the more aggressive forms of BC [24]. Such observations are corroborated from tissue microarray studies on a cohort of 45 BC cases with more than 80% being TNBC [16]. It was found that patients with high-grade tumors and positive lymph node status exhibit elevated levels of PAPP-A expression. Heightened PAPP-A level was predictive of poorer survival outcomes and an increased risk of disease recurrence. BC cell line assays validated the trend of increased PAPP-A leading to higher aggressiveness and the study found further significance of the PAPP-A/IGF axis in BC, demonstrating that motility can be altered by manipulating components of the IGF axis. PAPP-A was upregulated in EGF and hypoxia-induced epithelial-mesenchymal transition (EMT) and PAPP-A expression strongly presented with a mesenchymal phenotype in BC cell lines as well as patient samples [16].

**Fig. 2:**
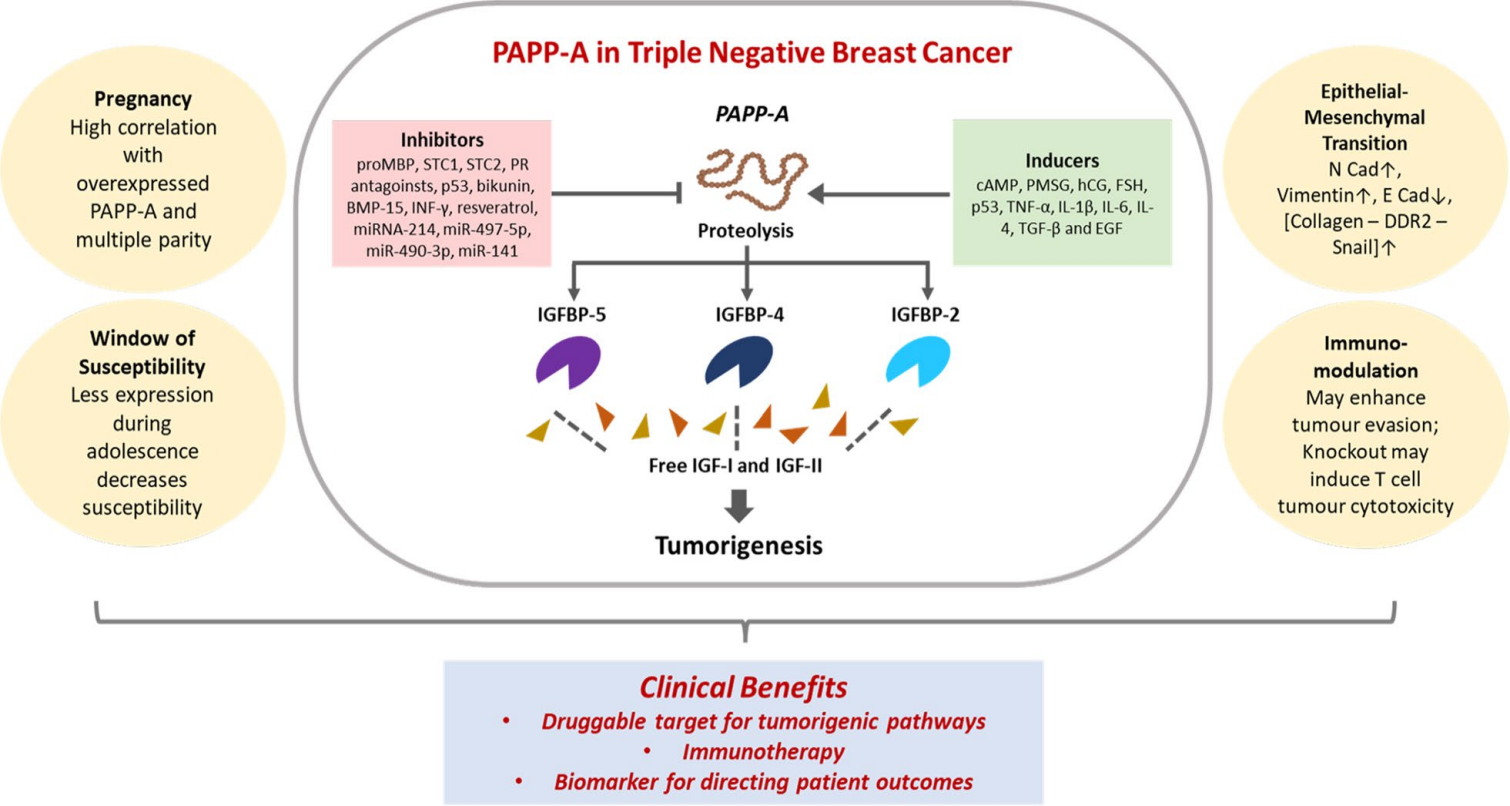
PAPP-A impacts pregnancy, epithelial-mesenchymal-transition, window of susceptibility and immunomodulation – representing potential clinical targeting of TNBC

PAPP-A is considered to have oncogenic activity in BC due to its impact on proteins of the IGFBP family (Table 3). With some exceptions, the binding proteins are regarded as generally tumor suppressive due to their inhibition of the mitogenic IGFs [88]. Numerous studies report increased IGFBPs lead to reduced aggressiveness of various cancers such as those of bladder, melanoma, lung and gastric in addition to BC. For instance, in bladder cancer, transcriptomic analysis of 200 patients and five normal samples revealed IGFBP-5 as a tumor sensitivity predictor of anti-IGF therapy that inversely corelated with IGF phosphorylation pathways [89]. In melanoma, an analysis of 54 melanoma cell lines by whole genome microarray expression profiling revealed upregulation of IGF-receptors (IGF-IR and IGF-IIR) and IGF-R substrates (IRS1 and IRS2) [90]. IGFBP-4 and-6 were also seen to be differentially expressed, with higher levels corresponding to more mesenchymal phenotypes. In the same study, 47 patient tumor samples were assessed, and PAPP-A expression was noted in 87% of metastatic tumor cases [90]. In melanoma cell lines and xenograft models, overexpression of IGFPB-5 also led to significant inhibition of malignancy through suppression of EMT, reduction of IGF signaling and decreased stem cell markers [91]. IGFBP-5 has been shown to promote p53 upregulation and resultant tumor suppression in gastric cancers. In the mammary gland, the IGFBP/IGF axis is heavily implicated in development and physiology of the breast; with its disruption playing a causal role in breast tumorigenesis [92, 93]. Out of the six IGFBPs known, IGFBP-5 is of particular relevance in BC [94, 95]. For instance, SNPs of IGFBP-5 is linked to increased risk of mammary tumors [96]. The multifunctional role of IGFBP-5 in apoptosis, migration, growth, cellular interactions and trafficking has been reviewed to reveal significant involvement in BC [94]. At present, proteolytic modulation of the IGFBP-5, -4 and -2 axis forms the basis of the known functions of PAPP-A activity in tumorigenesis (Fig. 2). In the following sections, we provide a comprehensive overview focusing on BC, especially the aggressive form of TNBC, with respect to the interplay between PAPP-A and the IGFBPs, where its role in promoting metastasis through EMT, impact on the window of susceptibility (WOS) as well as its influence in pregnancy associated oncogenesis is examined. PAPP-A mediated immunomodulation is emerging. However, while its immune relationship specifically in BC has not yet been studied, promising results from other cancers provide insights for BC cases. The clinical relevance of PAPP-A is subsequently reviewed, with several reports demonstrating it as a biomarker for disease progression, as well as a possible therapeutic target, and thus, the potential of targeting PAPP-A in providing an overall beneficial outcome is critically assessed and revealed (Fig. 2).

**Table 3:** Role of PAPP-A and IGFBP/IGF axis in BC

IGFBP/IGF	Role	PAPP-A expression	Organism	References
IGFBP-4 proteolysis	Leads to increase in IGF-I	Secreted along with IGF-I	Bovine mammary fibroblast cells	[98]
PAPP-A resistant IGFBP-4	Leads to sequestering of IGF-I	Retained tumor suppression; decreased angiogenesis and lung metastasis	4T1.2, orthotopic model of 4T1.2 BCs	[18, 99]
IGFBP-4 proteolysis	Due to increased PAPP-A levels from Skp2B overexpression	Putative p53 binding sites in PAPP-A gene	During pregnancy and lactation in the mice mammary glands	[81]
IGFBP-4	Co-expression with PAPP-A	Co-expression with IGFBP-4	HCC70, MDA-MB-468 and MDA-MB-231 cells	[16]
IGF-IR	Co-expression with PAPP-A	Co-expression with IGF-IR	HCC70, MDA-MB-468, HCC1954 and MDA-MB-231	[16]
IGF-IIR	Independent of PAPP-A expression	Expression with and without IGF-IIR	With PAPP-A: MDA-MB-468, HCC1954 and MDA-MB-231 Without PAPP-A: MCF-7, BT474, SKBR3, HCC1569, MDA-MB-453	[16]
IGFBP-5 proteolysis	Due to increased PAPP-A levels from increased collagen deposition	Higher in parous mice breast than in nulliparous mice	Transgenic mice overexpressing PAPP‐A in the mammary gland and MCF-7 BC cells	[102, 103]

### PAPP-A in TNBC: proteolysis of IGFBPs

Proteolysis of the IGFBPs by PAPP-A is a critical component of BC development and progression. The clinical correlation between BC cases and PAPP-A is demonstrated in case–control studies on individuals enrolled in a BC prevention program [97]. In the study, serum concentrations of total PAPP-A, IGF-I and IGFBP-4 were found to be possible indicators of BC prevalence in a normal population, with PAPP-A displaying remarkable differences between the control group and chemotherapy treated group [97]. Mechanistically, in breast tissue, the cleavage of IGFBP-4 by PAPP-A was revealed for the first time in bovine mammary tissues through in vitro immunoblotting assays. PAPP-A was identified as the protease which degrades IGFBP-4 to increase IGF-I availability in the bovine mammary fibroblast (BMF) cells, but not in bovine mammary epithelial (BME) cells, as none of the IGFBP regulatory components studied (IGF-I and IGF-II) could be detected in the BME cells [98]. Such variant expression could arise from differential regulation of IGFBPs in the bovine fibroblasts as compared to epithelial cells. In an orthotopic model of 4T1.2 BCs, it was seen that PAPP-A resistant IGFBP-4 retained their tumor suppressive role of sequestering IGFs even in the presence of PAPP-A, representing a novel avenue of IGF-blocking strategies for BC therapeutics [99]. This observation was further validated by utilizing a recombinant PAPP-A resistant IGFBP-4 in 4T1.2 cells to achieve reduction in cell migration and invasion through IGF-I suppression [18]. The recombinant IGFBP-4 could not be cleaved by PAPP-A and thus acted as an IGF-I reservoir to reduce bioavailability of the mitogen. Notably, direct intra-tumoral injection of the PAPP-A resistant IGFBP-4 decreased angiogenesis and lung metastasis in the 4T1.2 mice BC models [18].

The exceptionally high incidences of p53 mutations associated with aggressive BCs such as TNBC characterize one pathway leading to aberrant PAPP-A expression that can promote tumorigenic architecture in the breast. PAPP-A has been revealed to be the link between IGFBP-4 proteolysis and p53 defects in BC [81]. Skp2B is an F-box protein reported to be overexpressed in BC [100]. In transgenic mice with BC xenografts that overexpress Skp2B, a 4–fivefold increase in PAPP-A levels were seen in their breasts as compared to non-transgenic mice. This increase in PAPP-A correlated with reduced or loss in levels of IGFBP-4 during pregnancy and lactation in the mice mammary glands; indicating IGFBP-4 degradation. Furthermore, sequence analysis of the PAPP-A gene revealed putative p53 binding sites in intron 1 of the PAPP-A gene. Interestingly, overexpression of p53 in the p53-negative cell line MDA-MB-157 revealed that mutated p53 binds to the intron 1 site to activate PAPP-A transcription, whereas wild type p53 binding leads to PAPP-A suppression. Mutant p53 thus binds to activate PAPP-A which in turn cleaves IGFBP-4 to release IGFs that play a causal role in cancer etiology [81]. Such findings demonstrate the oncogenic activity of PAPP-A in BC that is mediated via the tumor suppressor p53. However, ChIP-seq analysis in other cell lines such as BJ/ET (human fibroblast foreskin cell line BJ immortalized by human telomerase reverse transcriptase) could not detect similar p53 binding sites in the PAPP-A gene. Instead, it revealed an overexpression in PAPP-A transcription by p53 mediated by other signaling pathways such as rat sarcoma (RAS) expression [82, 83]. Differences in cell type behaviors and the multifunctional mode of actions of p53 and PAPP-A, both of which require further elucidation, are major factors in accounting for the paradoxical effects seen with PAPP-A. PAPP-A is predominantly reported to possess oncogenic capacity [6], however that is also a matter of contention. The contradictory role is illustrated in BC cell lines and human mammary epithelial cells in which PAPP-A was found to have a tumor suppressive role [33]. Mitotic studies of FFPE specimens from a cohort of invasive BC patients as well as several BC cell lines revealed that a high proportion of luminal, HER2 and basal-like cases had PAPP-A silenced. Normal breast had much reduced silencing of PAPP-A and loss of function correlated with increasing progression of malignancy and higher invasiveness of BC [33]. In humans, a population-based cohort study including more than 600,000 pregnancies revealed that lower maternal serum concentration of PAPP-A in the first trimester appeared to bestow a higher long term BC risk [34]. Prenatal concentrations of alpha-fetoprotein (AFP), hCG, unconjugated estriol (uE3), PAPP-A, and dimeric inhibin-A (DIA) were also assessed. Only hCG, AFP, or PAPP-A were seen to indicate a slight elevation in future risk of hormone-dependent cancers such as BC [34]. However, the strongest outcome of the study was an overall lack in the future cancer predictive capacity of any of the assessed prenatal serum proteins, highlighting the strong need for further research for clarifying the mechanisms underlying some of the counterintuitive results seen with PAPP-A.

The pathogenic role of PAPP-A with respect to other IGFBPs such as IGFBP-2 has been noted but the underlying biological mechanisms with all IGFBPs are currently unknown. In a clinical evaluation of 301 females with BC and 531 non-cancer healthy controls, high PAPP-A level was predictive of worse prognosis for recurrence free survival (RFS) [101]. Serum levels of PAPP-A and IGFBP-2 independently prognosticated RFS and overall survival (OS), but no substantial increase in predictive value was observed. However, baseline quantities of PAPP-A and IGFBP-2 had no significant differences between cancer and non-cancer individuals. Interestingly, elevation of both PAPP-A and IGFBP-2 in serum was seen; suggesting IGFBP-2 proteolysis by PAPP-A occurs at a slower rate than IGFBPs-4 or 5 [101].

### PAPP-A in TNBC: role in epithelial-mesenchymal transition (EMT)

Epithelial-Mesenchymal Transition (EMT) is a vital cellular reprogramming process adopted by cancer cells for enhancing motility and migration in order to aid metastasis [104, 105]. PAPP-A/IGFBP/IGF axis is found to play a significant role in motility and EMT of TNBC. In a study evaluating PAPP-A mRNA expression through performing qRT-PCR assays in a panel of eight TNBC cell lines, high expression was reported in four cell lines (HCC70, MDA-MB-468, HCC1954 and MDA-MB-231) [16]. Of the cell lines expressing PAPP-A, three expressed IGFBP-4 (HCC70, MDA-MB-468 and MDA-MB-231), all four expressed IGF-IR, and eight cell lines expressed IGF-IIR independent of PAPP-A expression (MDA-MB-468, HCC1954 and MDA-MB-231, MCF-7, BT474, SKBR3, HCC1569, and MDA-MB-453). The role of PAPP-A/IGFBP axis in impacting the migratory ability of TNBC cells was subsequently revealed. Anti-PAPP-A antibodies were found to significantly reduce migration in MDA-MB-231 cells, as did anti-IGFPB-4 antibody. Co-culturing with anti-PAPP-A and anti-IGFBP-4 antibodies further reduced invasiveness; underscoring the pro-migratory impacts of PAPP-A and its involvement in EMT. The connection to EMT was validated and further explored by analyzing The Cancer Genome Atlas (TCGA) dataset of BC (n = 1105), as well as RNA sequencing, where PAPP-A significantly linked with co-expression of several canonical mesenchymal markers. Another dataset of 51 human BC cell lines revealed increased PAPP-A expression in basal B subtypes (corresponding to highest mesenchymal expressions) as compared to the more epithelial subtypes; thus, highlighting the tendency of PAPP-A to present with aggressive mesenchymal phenotypes of BC such as TNBC [16].

In another study, high PAPP-A expression was seen in BC cell lines including TNBC (T47D, MCF-7, BT549, and MDA-MB-231 and MDA-MB-468) as compared to normal breast epithelial cells MCF-10A through qRT-PCR and western blot assays [17]. Overexpression of PAPP-A in the cell lines MDA-MB-231 and MCF-7 significantly increased cellular proliferation, higher cell numbers in S phase of the cell cycle, and increased wound healing, migration and invasive capacity. Overexpression also led to higher protein levels of mesenchymal markers N-cadherin and vimentin and reduced epithelial marker E-cadherin. On culturing the above cell lines with PAPP-A rich serum collected from pregnancy associated BC patient samples, similar results were observed as those from PAPP-A overexpression. Conversely, knock down of PAPP-A using PAPP-A targeting siRNAs in the cell lines BT549 and MDA-MB-468 reduced proliferation, S phase cell numbers, wound healing, migration and invasive capacity; as well as reduced N-cadherin and vimentin and increased E-cadherin. miRNA regulation of PAPP-A was investigated, with miR-497-5p appearing to be a negative regulator of PAPP-A. The tumor promoting role of PAPP-A was also seen in vivo, where injection of PAPP-A recombinant protein in mice xenograft models led to significantly increased lung metastases [17].

IHC studies on the mammary glands of PAPP-A overexpressing transgenic mice, at the time of involution or postpartum, has identified a significant upregulation in Snail nuclear expression [103]. Snail oncogene is widely recognized as a key driver of EMT, with its nuclear expression being associated with the more aggressive malignant phenotypes [106]. On corroborating in MCF-7 cells overexpressing PAPP-A, significantly higher levels of Snail were similarly recorded, along with significant increase in invasive capacity, as compared to the non-PAPP-A overexpressing cells. Interestingly, the presence of collagen in the culture media further enhanced Snail expression in the PAPP-A overexpressing cells, as well as increasing levels of phosphorylated discoidin domain receptor 2 (DDR2), a known mediator of cancer progression that is a member of the collagen activation pathway [103, 107].

### PAPP-A in BC: role in the window of susceptibility (WOS)

The role of PAPP-A in WOS, i.e., time periods spanning childhood, adolescence and young adulthood for susceptibility to initiating events for long term BC, is emerging [108]. The mammary carcinoma susceptibility 5c (*Mcs5c)* locus on rat chromosome 5 shares homology with mice and humans [109]. PAPP-A regulation has been demonstrated in mammary glands of *Mcs5c* Wistar-Kyoto homozygous congenic rats and rat BC cell line LA7 and has been identified to be associated with the WOS leading to increased risk of BC development [110, 111]. Using chromosome conformation capture, the *Mcs5c* locus was found to bear a temporal control element that physically interacts with the neighboring PAPP-A gene located over 517 kb away in mammary epithelial cells. This interaction is genotype-independent and happens through an intriguing chromatin looping mechanism. A methylation interaction is also reported in addition to the looping, conversely occurring in a genotype dependent manner in vivo. Importantly, differential PAPP-A expressions were noted for two different WOS periods in the lifetime. Adolescent WOS was marked by the chromatin looping between *Mcs5c* locus and PAPP-A as well as significant methylation differences in the CpG regions which bear the PAPP-A looping fragment. In contrast, sexually immature WOS lacked the looping interactions and had no significant differences in the methylation patterns. The age-dependent changes of PAPP-A expression are of important note, indicating the significance of proteomic interactions occurring at specific developmental points; with decrease in PAPP-A levels during adolescence appearing to reduce susceptibility to BC [111].

### PAPP-A in TNBC: impact of pregnancy

Pregnancy, a condition featuring highly elevated levels of PAPP-A, marks another state that is linked with a short-term increase in risk of BC, and pregnancy related BCs present with higher rates of TNBC [112, 113]. In a population study of maternal and infant birth weights including more than 400 women, increased serum concentrations of PAPP-A and ratios of estriol/anti-estrogen alpha-fetoprotein were seen in females delivering heavier babies and was associated with a greater risk of BC [114]. Although in normal pregnancy, increased serum PAPP-A is mainly in the form of PAPP-A/proMBP complex, the elevated hormonal concentrations along with increase in bioavailable IGFs mediated by free PAPP-A levels presents the biological plausibility of PAPP-A creating a milieu favoring tumor development and growth.

The parallel between increased BC risk and PAPP-A expression during pregnancy is mechanistically linked. Involution of the breast, a key phenomenon in pregnancy and post-partum state, highly involves IGFBPs-4 and -5 and the altered breast architecture is rich in collagen [115–118]. Independent of the breast and/or pregnancy, PAPP-A is known to colocalize with newly synthesized collagen during wound healing of the skin [119]. Thus, the relationship between PAPP-A, collagen and BC is further striking. The oncogenic potential of PAPP-A was found to be pregnancy and collagen dependent in a study including transgenic mice overexpressing PAPP‐A in the mammary gland and MCF-7 BC cells [102]. Collagen deposition significantly upregulated PAPP-A expression during involution which led to increased IGFBP-5 proteolysis. The resultant elevation in IGFs signaled additional collagen deposition. PAPP-A also upregulated the La ribonucleoprotein domain family member 6 (LARP6) which further drove up collagen deposition [103]. Consequently, an oncogenic feedback loop between collagen and IGF signaling pathways via aberrant PAPP-A expression has been found. The loop was absent in mammary glands of virgin mice as they did not show PAPP-A mediated IGFBP-5 cleavage, indicating PAPP-A becomes tumor inducing only during pregnancy [102, 103]. IHC on tumor samples from 46 patients with BC revealed parous females (corresponding to 79%) had higher positive PAPP-A expression as compared to nulliparous females. Notably, this correlated with the parous group presenting with an increased incidence of TNBC as compared to the nulliparous group. Furthermore, multiple pregnancies increased the oncogenic capacity of PAPP-A [102]. This indicates the accumulation of PAPP-A associated factors following every pregnancy event – leading to an increase in risk window of BC. On the other hand, prolonged breast-feeding reduced BC risk by increasing the accumulation of PAPP-A inhibitory glycoproteins from the ovary, STC1 and STC2 [102].

Studying the tumor-associated collagen signature (TACS) of mice overexpressing PAPP-A in the mammary gland revealed that postpartum breasts have higher collagen signatures than virgin breasts, with the collagen being anti-tumorigenic in nature [103]. Of note was a particular orientation of collagen called TACS-3 that is associated with more aggressive forms of BC [120–122]. TACS-3 levels reduced and gave way to less aggressive collagen forms (TACS-1 and -2) in postpartum involution in non-transgenic mice, however in PAPP-A overexpressing transgenic mice, TACS-3 levels did not show reduction in postpartum involution [103]. The overexpression of PAPP-A post-partum thus transforms the collagen during involution from tumor inhibiting to tumor promoting TACS-3. In normal human breasts, PAPP-A is periodically expressed rather than constitutively [103]. The study reports that in human postpartum BC driven by PAPP-A, IGF signaling and TACS-3 collagen formation is constitutive. Furthermore, both in vivo transgenic mice and in vitro cell line studies in MCF-7 cells showed that one of the impacts of PAPP-A overexpression is on the DDR2/Snail pathways. PAPP-A activated DDR2 phosphorylation and Snail expression through promoting collagen production in an IGFBP proteolytic dependent manner. As explored in "PAPP-A in TNBC: role in Epithelial-Mesenchymal Transition (EMT)" section of this review, elevated phosphorylated DDR2 and Snail are known drivers of cancer progression and EMT; and experimentally correlated with significantly higher invasion and migration. In line with this, a CRISPR mediated deletion of DDR2 led to elimination of the invasion-promoting effects of PAPP-A. Furthermore, gene set expression analysis for screening human BC datasets revealed that BC patients showing positive PAPP-A, Snai1 and Col1A1 signatures presented with shorter OS and increased metastasis. Out of 13 significantly dysregulated pathways in the above genotype signature, EMT, extracellular matrix (ECM) architecture and collagen formation represented the most elevated pathways [103].

### PAPP-A in TNBC: in vivo studies on BC models

Utilizing an orthotopic model of 4T1.2 mice, Harmey et al. studied the role of PAPP-A mediated IGFBP-4/IGF1 axis in BC [18, 99]. A recombinant PAPP-A resistant IGFBP-4 (dBP4) was bioengineered, and it was seen that dBP4 retained the capacity to bind IGF-I through western blotting and surface plasmon resonance on initial in vitro studies in 4T1.2 mouse mammary adenocarcinoma cells. dBP4 acted as an IGF-I reservoir that could block IGF-I mediated Akt signaling. IGF-I preincubated with dBP4 reduced cell migration, invasion and angiogenesis. In line with the reduced bioavailability of IGF-I leading to anti-tumor effects, dBP4 alone did not impact migration or invasion. The purified dBP4 was then injected into the orthotopic BC model – 4T1.2luc cells implanted in the mammary fat pad of BALB/c mice. 4t1.2luc cells, transfected with luciferase to allow for in vivo imaging, and along with CD31 immunohistochemistry, revealed a reduction in angiogenesis and a decrease in lung metastasis in the dBP4 injected mice. The reported data demonstrates a direct link between PAPP-A resistance that leads to advantageous reduction in tumor angiogenesis and metastasis in BC [18, 99]. When complexed to IGFBP-4, IGF-I is non-mitogenic, but IGFBP-4 can be proteolyzed by PAPP-A to release active IGF-I.

PAPP-A and the IGFBP-4 /IGF-II axis in BC is shown to be p53 dependent in vivo [81]. Transgenic mice that overexpress Skp2B in the mammary glands develop breast tumors and other phenotypes such as increased side branching and pregnancy-like features in virgin females. Proteolytic cleavage of IGFBP-4 and defective p53 activity are a key feature of the breast tumors seen in the transgenic mice. The study reports that denatured p53 activates PAPP-A, which in turn cleaves IGFBP-4 and leads to a likely hyperactivation of IGF-II signaling. RT-PCR revealed a 4–fivefold increase in PAPP-A levels in the breast tissue of transgenic mice compared to non-transgenic mice. In addition, a transient increase in PAPP-A levels in the breast was seen during pregnancy that corresponded with decrease in IGFBP-4 levels in the mice during pregnancy and lactation. Thus, overexpression of Skp2B led to an increase in PAPP-A expression in the mammary glands during non-pregnant state as well. Mechanistically, the authors found Skp2B leads to formation of denatured p53 that binds to intron 1 of PAPP-A and enhances IGF-I and -II. In the absence of Skp2B, wild type p53 suppressed PAPP-A and led to reduced availability of IGF-II [81].

The upregulation of PAPP-A in pregnancy associated BC tissues and BC cells has been further reported in mice xenograft models subcutaneously injected with the TNBC cells MDA-MB-231 [17]. A significant increase in tumor growth, tumor weight and the number of metastatic lung nodules was seen in the mice upon injection with PAPP-A. This coincides with the reports from Harmey et al.whereby PAPP-A resistant IGFBP-4 led to reduced lung metastasis in mice – indicating the role of PAPP-A as an oncogenic factor for BC in vivo [17, 99].

PAPP-A is demonstrated to be a contributing factor that enhances the susceptibility of post-partum breasts to more aggressive cancers such as TNBC by impacting the breast collagen architecture [103]. Utilizing mice models overexpressing PAPP-A in the mammary gland in FVB/n background and xenograft mice models using MCF-7 and MCF-7 with overexpressed PAPP-A or KO PAPP-A, it is now known that PAPP-A transforms the anti-proliferative post-partum collagen into pro-tumorigenic collagen and elevates the well documented drivers of cancer progression and EMT, DDR2 and Snail [103]. Furthermore, studies on PAPP-A transgenic mice have revealed that extended lactation affords protection against tumor formation. The safeguarding impact of lactation is linked to the expression of PAPP‐A inhibitors, namely STC1 and STC2 [102].

Regulation of PAPP-A is reported to involve the Mcs5c locus and forms a major component of the cellular processes underlying breast development and the WOS period for BC disease pathology [111]. Congenic rat lines with the resistant Wistar-Kyoto (WKy) Mcs5c allele on a susceptible Wistar-Furth (WF) background have been utilized to identify the complex regulatory mechanism of PAPP-A expression during specific WOS that involves chromatin looping and DNA methylation. The interaction, discussed in detail in "PAPP-A in BC: role in the Window of Susceptibility (WOS)" section, signifies a protective advantage associated with reduced PAPP-A levels during adolescent development. Taken together, the results from animal studies tend to characterize PAPP‐A as a pregnancy-dependent oncogene in vivo, underscoring that diminished PAPP-A levels confer protective measures against aggressive BC initiation and progression.

## Immunological relevance of PAPP-A in BC

The immune system plays an indispensable role in cancer progression and resistance to therapy, with immune evasion being a common feature of tumor survival and metastasis in cancers such as those of the breast [123–125]. It is a multi-step process that is largely linked to the tumor-immune microenvironment, a suppressive cytokine milieu and the diminished ability of the innate and adaptive immune systems to effectively detect and eliminate tumor cells [126, 127]. These factors are key reasons why therapeutic strategies against cancers, especially TNBCs, remain troublesome. Increasingly, immunotherapy is becoming one of the most clinically relevant strategies for effective treatment in BC [128–131]. In the neonatal setting, PAPP-A maintains immune homeostasis. However, in oncogenesis, PAPP-A is associated with tumor initiation, migration and invasion – pathways that comprise the molecular and cellular events of EMT, and this reprogramming process is well known to be strongly related to immune evasion [8, 17, 24, 49, 90, 132, 133]. Importantly, PAPP-A levels have been reported to be directly elevated during EMT [134, 135]. Nevertheless, to the best of our knowledge, very little is known about the overlap between cancer, PAPP-A and immune evasion, with the link being unreported in BC. An initial correlation has so far been shown in Ewing sarcoma, followed by liver cancer, though the mechanisms remain unidentified.

As PAPP-A expression is high in Ewing sarcoma, a study investigating the profile of T cell receptor (TCR) transgenic T cells against PAPP-A revealed enhanced T cell targeting in A673 Ewing sarcoma cells and mice xenografts [132]. The study showed that the isolated T cell clone PAPP-A-2G6, which recognizes a specific PAPP-A peptide, was able to target A673 cells, and further, PAPP-A-specific T cells also lysed the cancer cells [132]. Functionally, the PAPP-A specific T cells harbored both a central memory and effector memory phenotype. Central memory phenotype facilitates T cell homing to secondary lymphoid organs and effector memory harbors a more cytolytic phenotype [136]. The findings were validated in vivo where immune deficient Rag2-/- γc-/- mice on a BALB/c background, inoculated with A673 tumor cells to form xenografts, were injected with a combination of PAPP-A-2G6 TCR transgenic T cells and CD8 + depleted peripheral blood mononuclear cells (PBMC), along with relevant controls [132]. The results indicated an enhanced tumor elimination process, lower tumor burden, greater accumulation of infiltrating CD8 + T cells in the tumor, while paired with no adverse effects in mice given PAPP-A-2G6 TCR transgenic T cells. Notably, IHC showed that PAPP-A expression in the A673 xenografts was higher compared to adjacent normal murine tissue, determining a correlation between PAPP-A expression, PAPP-A-2G6 TCR transgenic T cells and CD8 + T cell infiltration [132].

An RNA-seq study in Ewing sarcoma cell lines with PAPPA knockout demonstrated the induction of immune related genes and the downregulation of reactive oxygen species, DNA repair and the endoplasmic reticulum unfolded protein response [8]. The immune related genes included those that are part of the complement system, allograft rejection, inflammatory response, acute-phase response, tumor necrosis factor alpha signaling, and interferon responses [8]. Moreover, from this data set, important antigen processing/presentation pathway genes such as peptide loading complex TAP-1/TAP-2/TAPBP, the proteasome components low molecular mass polypeptides 2 and 7 (LMP2 and LMP7), proteasome activator complex subunits (PSME) 1 and 2, and the endoplasmic reticulum aminopeptidase 1 (ERAP1) were also found to be upregulated in PAPP-A knockout cells. Downregulation of antigen processing/presentation pathways are yet another way tumor cells evade detection by the immune system [132]. Of particular interest, genes associated with the subunits of major histocompatibility complex (MHC) class I molecules (beta2-microglobulin and classical MHC class I human leukocyte antigens A, B and C) were enriched, indicating that PAPP-A could potentially impact antigen processing and presentation machinery and the adaptive immunity as MHC molecules are heavily involved in the adaptive arm of the immune system [137]. MHC class I molecules are expressed on all nucleated cells and are essential for presenting foreign peptide antigens to activate cytotoxic T cells function to directly target and kill tumor cells [137, 138]. Further to this, in the absence of PAPP-A (EW8 cell line with PAPP-A knockout), allogenic T cells had enhanced killing capacity, supporting the notation that PAPP-A may support tumor progression via depleting the cytotoxic functions of T cells [8]. The use of such TCR transgenic approach targeting PAPP-A is thus suggestive of a potentially beneficial route of novel therapeutics in BC.

PAPP-A knockout profiles are not unique to Ewing sarcoma and may represent a general feature of cancer physiology. The downregulation of immune related genes reported by Heitzeneder et al. was also seen in livers of BL\6 PAPP-A overexpressed mice [139]. TCGA data was analyzed from a cohort of 361 individuals with hepatocellular carcinomas in order to determine the link between PAPP-A signature and immune evasion in humans. Consistent with the general theme of PAPP-A expression and the downregulation of immune profiles, there was a strong association of PAPP-A signature and a subgroup of hepatocellular carcinomas with an exhausted immune phenotype [139]. Both males and females were included in the cohort analyzed indicating that the PAPP-A signature is independent of gender. Overall, while the mechanistic basis remains uncertain, these studies suggest PAPP-A may alter the expression profile and phenotype of immune-related pathways leading to immune evasion.

## Clinical relevance of PAPP-A in TNBC

PAPP-A is an important component of regulating IGF availability through the IGFBP receptor axis; with several of the IGFs and IGF receptors acting as clinical biomarkers [140–143]. The potential of PAPP-A itself as a clinical marker is gradually emerging as highly promising, and is of particular focus in this review (Table 4). The co-incidence of PAPP-A with BC cases was revealed to be of clinical relevance as a significant predictor initially in predicting recurrence in stage II BC patients [87]. Immunoperoxidase based examination of PAPP-A, pregnancy specific β-1-glycoprotein, and placental protein five in primary tumors and metastases from 30 patients undergoing low- or standard-dose combination chemotherapy for stage II BC was evaluated in conjunction with 25 traditional clinicopathologic features in relation to early recurrence (within two years). Among the 11 patients experiencing early recurrences, nine (82 percent) tested positive for PAPP-A, whereas 16 out of the 19 remaining patients were negative. None of the other clinicopathologic features showed a correlation with early recurrence [87].

**Table 4:** PAPP-A as a clinical biomarker for BC

Clinical indicator	Sample Size	Analysis method	References
Independent predictor of early recurrence	Stage II BC: 30 cases (treated with low or standard chemotherapy)	Immunostaining and clinicopathology	[87]
Independent predictor of early recurrence	Stage I ER negative: 40 cases	Immunostaining and clinicopathology	[144]
Independent predictor of early recurrence; independent of ER status	Stage I ER positive: 30 cases	Immunostaining and clinicopathology	[23]
Worse prognosis seen in elevated PAPP-A; Independently prognostic for RFS and OS in the long-term	Early BC (with and without treatment): 301 cases, Non-cancer: 531 cases	Serum assays on patient samples	[101]
Predictor of malignancy presence; positive association with serum activin A, serum activin B, total IGFBP-4, and correlation with total IGF-I; negative association with total cholesterol and triglycerides	Benign tumors: 100 cases, Malignant BC (treatment naïve and chemotherapy): 145 cases Non-cancer: 100 cases	Serum assays on patient samples	[97]
High expression correlated with lymph node metastasis and high-grade tumor; worse prognosis, disease recurrence and poor OS in high-grade BC	BC: 45 cases (with 80% TNBC)	IHC on TMA	[16]
PAPP-A/SNAI1/COL1A1 expression panel: High score correlates with distant metastases	Primary BC: dataset of 327 cases	Gene set analysis	[103]
Silencing links with distant metastases	Invasive BC: 173 cases Normal breast: 30 cases	DNA methylation analysis on FFPE	[33]
Low serum level in first trimester: greater long-term BC risk	677,247 pregnancies	Biochemical screening	[34]

Subsequently, 40 cases with stage I ER negative BC were assessed for a range of 33 clinicopathological features and examined for immunostaining with PAPP-A, carcinoembryonic antigen, human chorionic gonadotrophin and pregnancy specific beta-1 glycoprotein [144]. PAPP-A staining (along with necrosis, nuclear atypia, and mitoses) significantly correlated with tumor recurrence in pairwise correlations. PAPP-A (along with extensive necrosis) also emerged as a significant independent predictor in 56% of the recurrent patients. In combination with extensive necrosis as the only other significant independent predictor found in the study, PAPP-A positivity was seen in 81% of the recurrent cases [144]. The study was extended to analyze 30 ER positive cases at stage I, assessed for a range of 25 clinicopathological features in addition to PAPP-A expression [23]. On immunostaining patient samples, PAPP-A positivity again emerged as significantly correlating with both ER positive (30%) or negative (40%) cases, as well as a significant independent predictor of early recurrence in ER positive (13.3%) or negative (20%) cases. PAPP-A thus possessed recurrence predictive capacity independent of ER status. Furthermore, comparison frequencies revealed no correlation of PAPP-A recurrence predictive capacity between ER or progesterone receptor (PR) status [23].

An intensive study comparison has been carried out for several IGFs, IGFBPs and PAPP-A levels between 301 patients (with early BC treated by surgery, with and without adjuvant treatments) and 531 non-cancer individuals as controls [101]. For some treatment regimens, statistical significance was seen in IGF-I, IGFBP-3 and PAPP-A between the cancer and non-cancer individuals, but biological relevance was reported as low. On evaluating RFS and OS of the BC groups, elevation in circulating IGFBP-2 and PAPP-A quantities in the serum, determined at start of treatment, was found to be independently associated with worse prognosis in females with long-term follow up subsequent to treatment. Interestingly, in contrast to previous studies with PAPP-A tissue staining, the study concluded serum PAPP-A as independently prognostic for RFS and OS in the long-term, but not in early recurrence of BC [101]. While such contrasting observations could arise from low sample sizes that are limitations of the studies, it is possible that the biological role of PAPP-A may be varied and differential in both cellular and temporal contexts of BC, further standing to reason serum, tissue and stage dependent correlations with PAPP-A.

The IGFBP-4/ IGF-I/PAPP-A axis along with follistatin-like (FSTL)-3 has been shown to be surrogate markers for BC in a study involving 100 females with benign tumors, 145 females with malignant tumors (including treatment naïve and chemotherapy treated) and 100 disease-free control individuals [97]. In adjusted comparisons, serum PAPP-A (along with FSTL-3, IGFBP-4, and IGF-I) was reported to have substantial elevation across groups between treated and non-treated samples, but statistical significance was not observed. Positive PAPP-A association was also seen with serum activin A, serum activin B, total IGFBP-4, and a correlation with total IGF-I. Interestingly, negative PAPP-A association was seen repeatedly with total cholesterol and triglycerides. Importantly, PAPP-A (as well as total IGF-I, total and intact IGFBP-4) was shown to be a predictor of the presence of malignancy and total IGFBP-4 independently positively corelated with PAPP-A [97].

The immunoreactivity of PAPP-A and its association with clinicopathological characteristics in BC has been evaluated in a study cohort of 45 BC patients [16]. IHC on tissue microarrays (TMA) of BC samples, comprising 80% cases as TNBC, revealed 57% as positive for cytoplasmic and membranous PAPP-A expression; with majority of PAPP-A expression seen for tumor tissues corresponding to T2 staging. Furthermore, elevated PAPP-A expression strongly tended towards high-grade tumors and correlated with involvement of lymph node status. While PAPP-A expression did not correlate with OS in the limited sample size, it strongly trended with worse prognosis (median survival of 25 months) as compared with PAPP-A negative expression (median survival of 69 months). Importantly, increasing the sample size to include nine datasets revealed elevated PAPP-A expression to link with high risk of disease recurrence, and significantly correlated with poor OS in grade 3 BC as compared to grades 1 or 2 [16].

In "PAPP-A in TNBC: impact of pregnancy" section, we review the association of collagen with PAPP-A in promoting aggressive BC. In line with the results discussed, the study analyzing the link between PAPP-A, collagen, and Snail used the gene expression panel (PAPP-A/SNAI1/COL1A1) to assess clinical outcomes of patients from a dataset of 327 cases with primary BC. In the survival analysis, the population scoring high in the panel significantly associated with distant metastases as compared to the low scoring group [103]. While the majority of studies have found PAPP-A expression or elevation to coincide with BC progression, some contradictions have been reported. A study investigating epigenetic silencing of FFPE cancer and non-cancer tissues found that PAPP-A is strongly silenced through promoter hypermethylation in 46% (80/173 cases) of invasive BC. The PAPP-A promoter was non-methylated in 90% (27/30 cases) of normal mammary tissues [33], indicating PAPP-A silencing to be associated with oncogenesis. In another report, the results from a population-based cohort study that looked at 677,247 pregnancies to assess long-term risk of cancer in females found lower levels of serum PAPP-A in the first trimester to correlate with greater risk of BC in the long-term [34]. However, the risk no longer appeared significant after adjusting for covariates. Greater risk was also seen with abnormally reduced PAPP-A levels in parous females as compared to nulliparous [34].

As is evident, further research on PAPP-A is required to tease out the underpinnings in order to reach a unified consensus. Nonetheless, utilizing PAPP-A in the clinical setting for BC exists with great promise. Given the documented evidence in literature highlighting its critical role in cell proliferation, exploring therapeutic strategies to target PAPP-A activity is thus crucial. PAPP-A expression has been revealed to be cell-specific, non-constitutive and responsive to external stimulus. Importantly, the presence of overall healthy longevity seen in PAPP-A KO mice supports the lack of adverse effects that can be expected on targeting PAPP-A [145]. This presents an avenue for optimizing selective targeting and, ideally, for teasing out cancer versus non-cancer phenotypes. The secretory nature of PAPP-A and its presence in extracellular tumor microenvironments can make it amenable to drug interventions. Reports predominantly support the focus on inhibiting PAPP-A proteolytic activity in a target specific manner. As such, the PAPP-A gene, mRNA and protein structures and regulation presents several sites and modes for therapeutic interventions – either directly with PAPP-A or with PAPP-A-interacting moieties. Notably, while lesser in number, the reports demonstrating conditional tumor suppressive milieu as a result of PAPP-A need to be further explored. Importantly for TNBC that has limited therapeutic options, the results with PAPP-A elucidate its potential in providing novel, improved or adjuvant strategies to improve outcomes.

## Conclusion

TNBC represents the most aggressive form of BC with the least favourable outcomes, are naturally recurrent, have poor prognosis and there is a steady increase in incidence rates [31]. Unlike other BC subtypes, dearth of efficient and optimal therapeutic options remain a major cause behind the dismal survival rates (approximately 10 months) seen in patients [31]. PAPP-A represents a promising clinical strategy for utilizing as a treatment modality and/or biomarker that ought to be exploited to address such critical unmet needs. Currently, the most evident method of PAPP-A mediated TNBC therapy appears to involve the IGFBP/IGF axis. Direct inhibition of IGF-I receptor utilizing antibodies have shown significant benefits only in early phase clinical trials, with a lack of similar efficiency seen in phase II–III trials due to insufficient specificity [146, 147]. Indirect blocking of the IGF receptors, utilizing therapies targeting PAPP-A, may thus be conceptualized as more selective or tissue specific than targeting IGF receptors. The role of PAPP-A in addition to proteolysis is as of yet unknown, but cannot be ruled out. Furthermore, it is clear that PAPP-A is heavily involved in p53 mediated pathways, collagen deposition, EMT and ECM tissue remodeling of BCs including TNBC – representing a clear benefit of PAPP-A targeting to impact such tumorigenic pathways. The majority of research till date presents PAPP-A as a cancer promoting antigen. However, the possibility of PAPP-A correlating with tumor suppression has been reported, highlighting the need for further research. Immunotherapy has been of great benefit in several cancers that have limited therapeutic targets such as TNBC, and PAPP-A expression has been shown to influence immune cells and immune related pathways. As TNBC is a difficult cancer to target due to its lack of ER, PR and HER2 expression, shedding light on the relationship and mechanisms between PAPP-A and the immune system may potentially lead to successful immunotherapeutic targets. In addition, quantifying PAPP-A levels can potentially contribute to positive patient outcomes by influencing patient selection and directing more accurate therapy regimens. In cases with aberrantly overexpressed PAPP-A, intervention at the prevention stage may also be a viable option. Through more focused research, a comprehensive and integrated understanding of the PAPP-A mediated impacts in TNBC is thus needed, representing a critical step forward in providing favorable outcomes and reliable clinical benefits.

## Data Availability

Not applicable.

## Abbreviations

5’ UTR: 5’ Untranslated region
AFP: Alpha-fetoprotein
BC: Breast cancer
BME: Bovine mammary epithelial
BMF: Bovine mammary fibroblast
BMP: Bone morphogenetic protein
cAMP: Cyclic adenosine monophosphate
CCP: Complement control protein
ChIP-seq: Chromatin immunoprecipitation followed by sequencing
DDR2: Discoidin domain receptor 2
DIA: Dimeric inhibin-A
ECM: Extracellular matrix
EGF: Epidermal growth factor
EMT: Epithelial-mesenchymal transition
ER: Estrogen receptor
ERAP1: Endoplasmic reticulum aminopeptidase 1
FFPE: Formalin-fixed paraffin-embedded
FSH: Follicle stimulating hormone
FSTL: Follistatin-like
hCG: Human chorionic gonadotropin
HER2: Human epidermal receptor 2
IGF: Insulin-like growth factor
IGFBP: Insulin-like growth factor dependent insulin-like growth factor-binding proteins
IGF-IR: Enhanced type I insulin-like growth factor receptor
IGF-IIR: Enhanced type II insulin-like growth factor receptor
IHC: Immunohistochemistry
IL-1β: Interleukin 1 beta
LARP6: La ribonucleoprotein domain family member 6
LMP: Low molecular mass polypeptides
LNR: Linear notch repeat
Mcs5c: Mammary carcinoma susceptibility 5c
MHC: Major histocompatibility complex
miR: MicroRNA
OS: Overall survival
PAPP-A: Pregnancy associated plasma protein-A
PBMC: Peripheral blood mononuclear cells
PMSG: Pregnant mare serum gonadotropin
PR: Progesterone receptor
proMBP: Proform of the eosinophil major basic protein
PSME: Proteasome activator complex subunits
qRT-PCR: Real-time quantitative reverse transcription pcr
RAS: Rat sarcoma
RFS: Recurrence free survival
SNP: Single nucleotide polymorphism
STC: Stanniocalcins
TACS: Tumor-associated collagen signature
TCGA: The cancer genome atlas
TCR: T cell receptor
TGF-β: Transforming growth factor beta
TMA: Tissue microarrays
TNBC: Triple negative breast cancer
TNF-α: Tumor necrosis factor alpha
uE3: Unconjugated estriol
WOS: The Window of Susceptibility
